# The complete mitochondrial genome information of *Hynobius unisacculus* (Amphibia, Caudata, Hynobiidae) and the phylogenetic implication

**DOI:** 10.1080/23802359.2019.1679680

**Published:** 2019-10-21

**Authors:** Ho Young Suk, Dong-Young Kim, Sunho Cha, Mi-Sook Min

**Affiliations:** a Department of Life Sciences, Yeungnam University, Gyeongsan, Gyeongsangbuk-do, South Korea;; b GenoTech Corporation, Daejeon, South Korea;; c Research Institute for Veterinary Science, College of Veterinary Medicine, Seoul National University, Seoul, South Korea

**Keywords:** *Hynobius unisacculus*, Hynobiidae, Korean Peninsula, phylogeny

## Abstract

*Hynobius unisacculus* is a hynobiid salamander species found only in a limited area at the southernmost part of the Korean Peninsula. Here, we characterized the complete mitochondrial genome of this species that was used to identify the phylogenetic relationship with other *Hynobius* species. The whole sequence was 16,411 bp and included 13 protein-coding genes, 2 ribosomal RNA genes, and 22 transfer RNA genes. The gene arrangement was completely identical to those observed in other *Hynobius* species. Upon robust phylogenetic tree reconstructed based on 13 protein-coding genes, *Hynobius* species living on the Korean Peninsula showed close phylogenetic affinity; *H. unisacculus* and *H. quelpaertensis* formed a cluster that was the sister to the cluster of *H. leechii* and *H. yangi*.

Over two decades, several new *Hynobius* salamander species have been discovered on the Korean Peninsula, a very small area on the southern tip of East Asia (Kim et al. 2007; Baek, Lee, Lee et al. 2011a; Baek, Lee, Song et al. 2011b; Min et al. 2016). *Hynobius unisacculus* is the fourth *Hynobius* species officially recorded on this peninsula after *H. leechii*, *H. quelpaertensis* and *H. yangi* (Baek, Lee, Lee et al. 2011a; Baek, Lee, Song et al. 2011b; Min et al. 2016). Upon a phylogenetic tree reconstructed based on cyt *b* and 12*S r*RNA, this species formed a single cluster with *H. quelpaertensis* among these three previously known *Hynobius* species (Baek, Lee, Lee et al. 2011a; Baek, Lee, Song et al. 2011b; Min et al. 2016). *Hynobius unisacculus* is found only in limited areas (Naro Islands, Goheung-gun, Suncheon-si, and Boseong-gun, Jeollanam-do, South Korea) at the southernmost part of the Korean Peninsula (Min et al. 2016). This species has not yet been designated as a legal protection target in South Korea. The phylogenetic structure of this species can thus provide important data for estimating the biogeographical pathways on the Korean Peninsula and establishing the management strategies.

We characterized the complete mitochondrial genome of this species to identify the robust phylogenetic relationship with other *Hynobius* species. The DNA sample was isolated from an ethanol-immersed specimen stored in the Conservation Genome Resource Bank for Korean Wildlife (CGRB: http://www.cgrb.org/) that was collected (34.438139, 127.478556) in 2010. The specimen is accessible as cgrb15732 in CGRB. Mitogenomic sequences were extracted using the CLC genomics workbench 6.5 (http://www.qiagenbioinformatics.com/) from the reads (5,406,233,748 bp) generated from the MiSeq platform. Each mitochondrial region was annotated using the MITOS web server (Bernt et al. 2013) and manually checked using the mitochondrial information of *H. leechii* (Zhang et al. 2006). The sequence information was deposited at NCBI GenBank under the accession number of MN242821.

The whole sequences were 16,411 bp and included 13 protein-coding genes, 2 ribosomal RNA genes, and 22 transfer RNA genes. L-strand was observed in eight tRNA genes and *ND6*. Every protein-coding gene contained ATG start codon with a single exception in *COX1* starting with *GTG. ND1, COX1, APT8, APT6, ND3*, and *ND4L* were terminated by *TAA, ND1,* and *COX2* by TAG, and *ND6* by AGA. Incomplete stop codon was detected at *COX3* (ATA), *ND4* (CAT), *ND5* (TTA) and *Cyt b* (ATA). The gene arrangement was completely identical to those observed in other *Hynobius* species (Zhang et al. 2006). Based on 13 protein-coding genes, we examined the phylogenetic placement of this species in the genus *Hynobius* (Figure 1). Korean species showed close phylogenetic affinity; *H. unisacculus* and *H. quelpaertensis* formed a cluster that was the sister to the cluster of *H. leechii* and *H. yangi* (Figure 1). Our study is predicted to be useful to reconstruct the consensus phylogenetic tree that is essential for the future study of biogeographic dispersal of terrestrial animals on the Korean Peninsula, which also can provide an important guideline to establish the management strategies for *Hynobius* species conservation.

**Figure 1.**
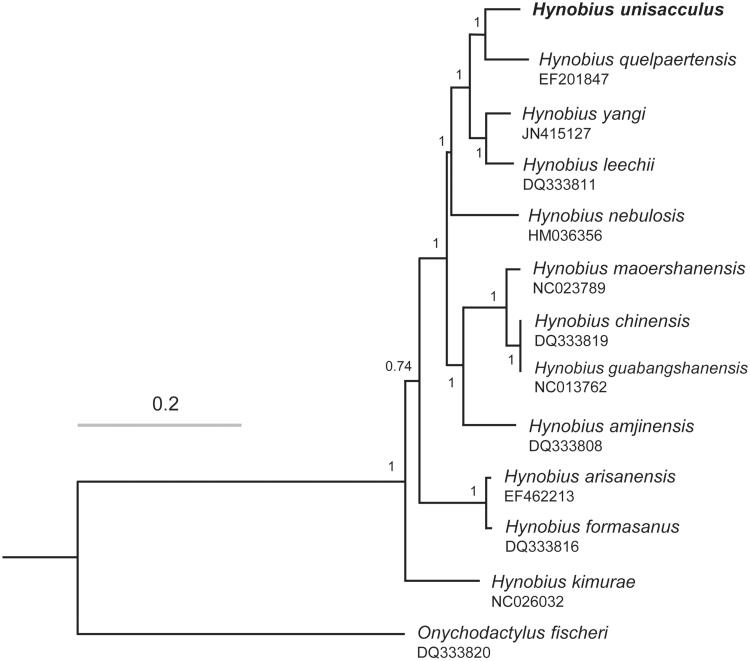
Bayesian inference tree of the genus *Hynobius* reconstructed by MrBayes 3.2 (Ronquist et al. 2012) using 13 protein-coding genes of *H. unisacculus* (bold) and 11 *Hynobius* species extracted from our analysis and NCBI GenBank. *Onychodactylus fischeri* in the same family was used as outgroup. *GTR + I+G* was selected as the best-fit substitution model by jModeltest 2.1.4 (Darriba et al. 2012) under Akaike information criterion (Akaike 1974), and two parallel runs were performed for one million Markov Chain Monte Carlo (MCMC) generations with sampling every 1000 steps. Posterior probabilities were indicated on the nodes.
